# The Patterns of Morphological Change During Intracerebral Hemorrhage Expansion: A Multicenter Retrospective Cohort Study

**DOI:** 10.3389/fmed.2021.774632

**Published:** 2022-01-13

**Authors:** Chang Jianbo, Xiao Ting, Chen Yihao, Wang Xiaoning, Shang Hong, Zhang Qinghua, Ye Zeju, Wang Xingong, Tian Fengxuan, Chai Jianjun, Ma Wenbin, Wei Junji, Feng Ming, Jianhua Yao, Wang Renzhi

**Affiliations:** ^1^Department of Neurosurgery, Peking Union Medical College Hospital, Peking Union Medical College and Chinese Academy of Medical Sciences, Beijing, China; ^2^Tencent AI Lab, Shenzhen, China; ^3^Department of Computer Science and Technology, Harbin Institute of Technology, Harbin, China; ^4^Department of Neurosurgery, Shenzhen Nanshan Hospital, Shenzhen, China; ^5^Department of Neurosurgery, Dongguan People's Hospital, Dongguan, China; ^6^Department of Neurosurgery, Linyi People Hospital, Linyi, China; ^7^Department of Neurosurgery, Qinghai Provincial People's Hospital, Xining, China; ^8^Department of Neurosurgery, Zhangqiu People Hospital, Jinan, China

**Keywords:** intracerebral hemorrhage, hemorrhage expansion, anatomy, shape, stroke

## Abstract

**Objectives:** Hemorrhage expansion (HE) is a common and serious condition in patients with intracerebral hemorrhage (ICH). In contrast to the volume changes, little is known about the morphological changes that occur during HE. We developed a novel method to explore the patterns of morphological change and investigate the clinical significance of this change in ICH patients.

**Methods:** The morphological changes in the hematomas of ICH patients with available paired non-contrast CT data were described in quantitative terms, including the diameters of each hematoma in three dimensions, the longitudinal axis type, the surface regularity (SR) index, the length and direction changes of the diameters, and the distance and direction of movement of the center of the hematoma. The patterns were explored by descriptive analysis and difference analysis in subgroups. We also established a prognostic nomogram model for poor outcomes in ICH patients using both morphological changes and clinical parameters.

**Results:** A total of 1,094 eligible patients from four medical centers met the inclusion criteria. In 266 (24.3%) cases, the hematomas enlarged; the median absolute increase in volume was 14.0 [interquartile range (IQR), 17.9] mL. The initial hematomas tended to have a more irregular shape, reflected by a larger surface regularity index, than the developed hematomas. In subtentorial and deep supratentorial hematomas, the center moved in the direction of gravity. The distance of center movement and the length changes of the diameters were small, with median values of less than 4 mm. The most common longitudinal axis type was anterior–posterior (64.7%), and the axis type did not change between initial and repeat imaging in most patients (95.2%). A prognostic nomogram model including lateral expansion, a parameter of morphological change, showed good performance in predicting poor clinical outcomes in ICH patients.

**Conclusions:** The present study provides a morphological perspective on HE using a novel automatic approach. We identified certain patterns of morphological change in HE, and we believe that some morphological change parameters could help physicians predict the prognosis of ICH patients.

## Introduction

Spontaneous intracerebral hemorrhage (sICH) produces mortality or disability in approximately 50% of cases (1, 2), imposing a severe burden (3, 4). Hemorrhage expansion (HE) occurs in approximately one-quarter of sICH patients (2, 5) and is a major determinant of deterioration and death (5–7). Thus, it is important to explore the changes associated with HE (8, 9).

In contrast to the volume changes (5, 10), little is known about the morphological changes that occur in HE (11). Some studies have shown that hemorrhagic lesions expand asymmetrically and non-uniformly, especially in the hyperacute phase (12, 13). However, the patterns of morphological change have not been explored. Additionally, many studies have found that the initial shape of a hematoma was associated with the quality of outcomes (14, 15). However, few studies have focused on the relationship between morphological changes and patient prognosis.

In the current study, we developed and applied a novel approach to explore the patterns of morphological change during HE, which provided a new perspective on hematoma expansion and might help physicians predict the prognosis of ICH patients.

## Methods

### Subjects

All data were obtained from the Chinese Intracranial Hemorrhage Image Database (CICHID), which was initiated by Peking Union Medical College Hospital (PUMCH) in February 2019 and supported by the Group of Medical Data, Chinese Medical Doctor Association (16). As of October 2020, the database contained approximately twenty-eight thousand scans from eight thousand patients at twenty-two centers located in Mainland China. All medical records and CT images were anonymized. The CT scans were in Digital Imaging and Communications in Medicine (DICOM) format.

The inclusion criteria were as follows: 1. The cohort from each center included more than 100 patients; 2. the medical records were searchable through the case retrieval system in each center; 3. the patients were adults diagnosed with spontaneous intracerebral hemorrhage (ICH); 4. the patients had one initial and at least one repeat non-contrast computed tomography (NCCT) scan not preceded by surgery; and 5. the initial CT scan was taken within 24 h after symptom onset, and the repeat scan was taken more than 8 but less than 72 h after the initial scan. The exclusion criteria were as follows: 1. The patients were diagnosed with secondary intracranial hemorrhage, such as epidural hemorrhage, subdural hemorrhage, traumatic brain injury, brain tumor, or hemorrhagic transformation of ischemic infarction; 2. the medical records were not available; 3. the hematoma volume in the repeat CT scan was <3 mL or the volume had decreased by more than 3 mL; and 4. the scans were low-quality images or failed to be registered to the atlas.

The boundary of each hematoma was determined on CT axial slices by a semiautomatic method, in which research assistants independently used the software platform ITK-SNAP 3.6 (17) to correct the boundary drawn by the laboratory's in-house automatic hematoma segmentation software (18). The following clinical characteristics were collected: age, sex, symptom onset time, Glasgow Coma Scale (GCS) score, Glasgow Outcome Scale (GOS) score, initial hematoma volume, location, intraventricular hemorrhage (IVH), and expansion. Both the hematoma boundary and the clinical characteristics were assessed by researchers (WXN, CYH and SH), and any disagreement was reviewed by a neurosurgeon (CJB). Absolute change and relative change were used to describe the change in hematoma volume, and HE was defined as an increase of at least 6 mL or 33% (5, 10).

### Measurement of Changes in Hematoma Morphology

The shape irregularity of each hematoma was measured by the surface regularity (SR) index (19, 20), calculated as follows: SR index = π^1/3^(6V)^2/3^/A, where V represents the volume and “A” represents the surface area of the hematoma. The SR index ranges on a continuous scale from 0 (very irregular shape) to 1 (perfectly regular sphere) (21).

Hematoma morphology was characterized in the initial CT by three diameters determined on the slices with the maximum hematoma area in the planes parallel to the coordinate system; these diameters are presented as length (anterior–posterior, AP), width (left–right, LR) and height (superior–inferior, SI) (Supplementary Figure 1) (22). The longitudinal axis of each initial hematoma was categorized into one or four types: AP, LR, SI, or no longitudinal axis (NL), determined by which diameter was the longest. The NL group was defined by pairwise ratios ranging between 0.850 and 1.176 for all pairs of diameters, which means that all three diameters were similar (Supplementary Figure 2).

The changes in diameters between the initial and repeat scans were described by the length change and direction change. The length change was calculated as the difference between the diameters of the hematoma on the initial and repeat scans. The direction change of the diameters was defined by the axis that showed the largest absolute change in length, categorized as AP, LR, SI or no direction change (Figure 2). “No direction change” was defined by similar length changes in two directions or in all three directions.

The geometric center was defined as the centroid of the largest connected region of the hematoma in 3D space. The distance of center movement was defined as the spatial distance between the geometric center locations of the hematoma on the initial scan and the repeat scan. The direction of center movement is presented as the projection of the vector's direction in the standard planes (axial, coronal and sagittal). The directions of center movement from all cases in the same anatomical region were synthesized into one arrow and visualized in an atlas (ICBM 2009c Non-linear Symmetric template, MNI152) (23) (Supplementary Figure 3).

### Image Processing

To compare morphological changes among different cases, the paired CT scans were registered to an atlas. After the skull was stripped away and the brain was extracted using BET (24), each pair of initial and repeat CT scans from the same patient was spatially registered to a common atlas using the MNI152 template (23, 25). The registration pipeline consists of two sequential linear registrations and two sequential non-linear registrations, where both registration tools were provided by Advanced Normalization Tools (ANTs), and the non-linear registration was based on a B-spline function (26). The Dice coefficient between the registered CT and the template was calculated after each registration. If the Dice value did not reach the predetermined threshold (0.93), the registration was considered a failure, and the pair of scans was excluded. To ensure the registration quality, a predetermined threshold was applied by visually checking the registered CT quality. After registration to a common atlas, all CTs and their hemorrhage masks were in the same template space. The synthesis to determine the direction of center movement was performed by the package Mayavi (27). The surface area, volume, geometric center and diameters were calculated by the image processing package Skimage (28).

### Prediction Model for Poor Outcomes (GOS ≤ 3) at Discharge

To explore the clinical significance of changes in hematoma morphology, we conducted multivariate logistic regression incorporating clinical parameters and hematoma morphological change parameters. Multiple logistic regression was used to select the most useful predictive variables for poor outcomes (GOS ≤ 3) at discharge. All useful predictors, defined as those with *P* < 0.05, were used to develop the final multivariate logistic regression, and a nomogram was then built to predict which ICH patients could have poor outcomes (29). Discrimination was evaluated by the area under the curve (AUC) value of the receiver operating characteristic curve (ROC), and calibration was measured by the calibration curve (30).

### Statistical Analysis

Baseline characteristics were summarized as counts [percentages (%)] for categorical variables and the mean [standard deviation (SD)] or median [interquartile range (IQR)] for continuous variables. Both clinical and morphological change parameters were investigated by descriptive analysis and difference analysis in subgroups (expansion and longitudinal axis type). A two-sided Pearson's chi-squared test or Fisher's exact test was used for categorical variables, and Student's *t*-test, ANOVA or the Mann-Whitney U test was used for continuous variables. The threshold for statistical significance was set to 0.05. Statistical analyses were conducted with SPSS Statistics (version 21.0.0, IBM, Armonk, New York) and R (version 3.6.3, R Foundation for Statistical Computing, Vienna, Austria).

## Results

**Figure 1:**
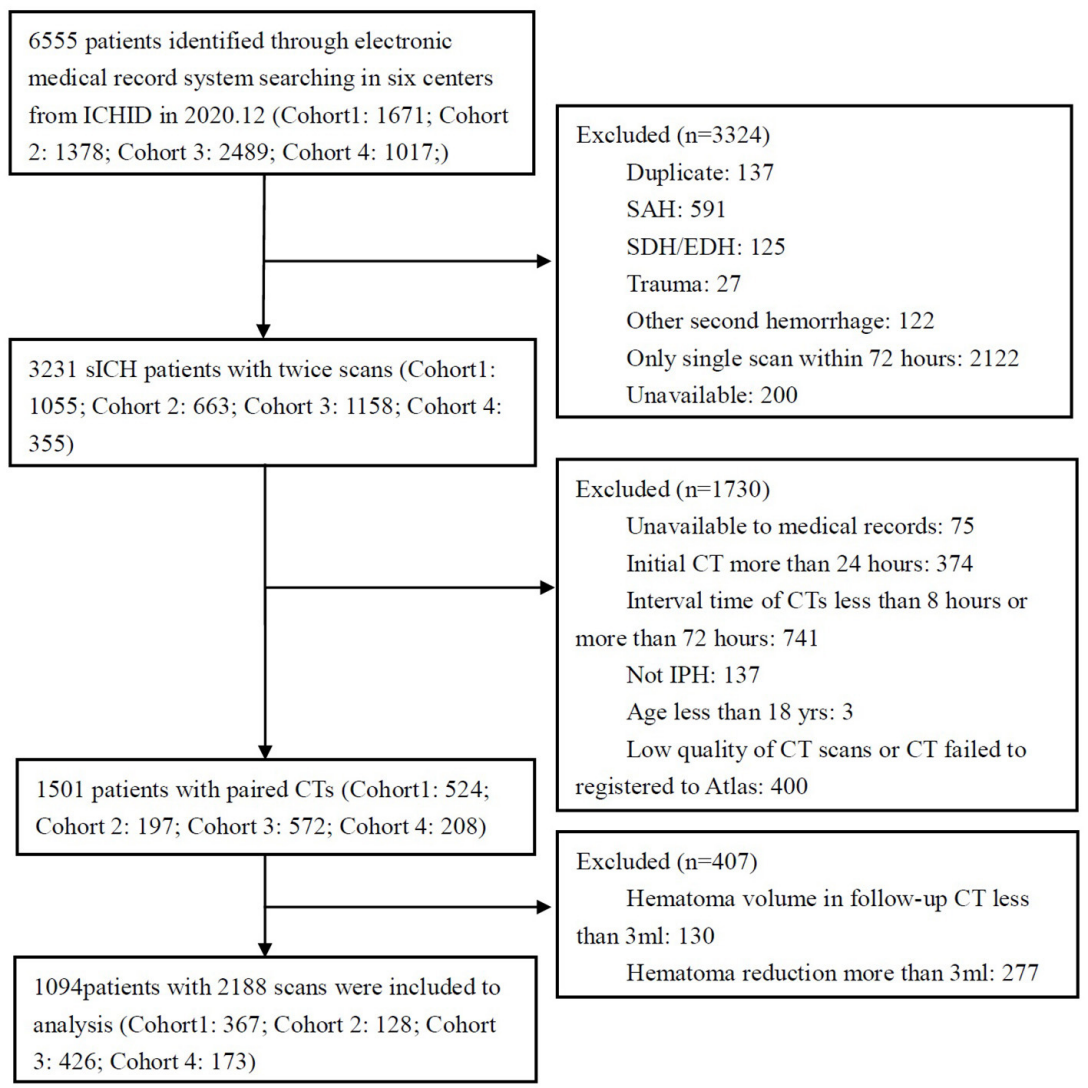
Flowchart of patient selection.

The database contained 3,231 ICH patients who underwent repeat CT between Jan. 1, 2016, and Aug. 30, 2020, at 4 medical centers; 1,094 of these patients met the inclusion criteria to be analyzed (Figure 1, Supplementary Table 1). The baseline characteristics of the patients are summarized in Table 1. Most included patients were elderly males with ICH in deep supratentorial brain regions. HE occurred in 24.3% of patients, with median absolute and relative increases of 14.0 (IQR 17.9) mL and 53.4 (IQR 97.3) %, respectively. The initial volume in the expansion group was larger than that in the non-expansion group (25.9 vs. 18.7 mL, *P* < 0.05). To compare the morphological changes, the CTs were registered to an atlas, as shown in Supplementary Figure 4.

**Table 1:** Baseline characteristics of ICH patients.

**Variable**	**Total** ** (*N* = 1,094)**	**Expansion group** ** (*N* = 266)^**a**^**	**Non-expansion group** ** (*N* = 828)**	***P*-value**
Male, *n* (%)	698 (63.8)	195 (73.3)	503 (60.7)	^*^
Age, median (IQR), y	61.0 (18.0)	59.0 (19.0)	61.0 (18.0)	
Onset to CT, median (IQR), hr	3.0 (4.0)	3.0 (3.0)	3.0 (5.0)	^*^
Time interval between CT scans, median (IQR), hr	22.8 (19.8)	22.4 (20.2)	22.9 (20.0)	
GCS score, median (IQR)	14 (4)	13 (5)	14 (3)	^*^
GOS score, median (IQR)	3 (1)	3 (1)	3 (1)	^*^
Initial hematoma volume, median (IQR), mL	20.2 (25.4)	25.9 (33.2)	18.7 (23.0)	^*^
IVH, *n* (%)	374 (34.2)	91 (34.2)	283 (34.2)	
Hematoma location				^*^
Deep, *n* (%)	763 (69.8)	176 (66.2)	587 (71.0)	
Lobar, *n* (%)	236 (21.6)	75 (28.2)	161 (19.5)	
Subtentorial, *n* (%)	94 (8.6)	15 (5.6)	79 (9.5)	
Absolute change in hematoma volume, median (IQR), mL	0.9 (5.5)	14.0 (17.9)	0.4 (2.4)	^*^
Percentage change in hematoma volume, median (IQR), %	4.5 (24.4)	53.4 (97.3)	6.8 (12.4)	^*^

a *Expansion was defined as a volume change ≥ 6 mL or 33%*.

* *P < 0.05. IQR, interquartile range; CT, computed tomography; GCS, glasgow coma scale; GOS, glasgow outcome scale; IVH, intraventricular hemorrhage*.

The morphological characteristics of HE are shown in Table 2. The hematomas became more irregular in repeat CTs in both the expansion and non-expansion groups, with the median SR index decreasing from 0.542 to 0.515. The median change in the SR index was−0.027, and the change in this index was significantly larger in the expansion group (-0.049) than in the non-expansion group (-0.020, *P* < 0.05).

**Table 2:** Morphological characteristics of hematoma expansion.

	**Total** ** (*N* = 1,094)**	**Expansion** ** (*n* = 266)**	**Non-expansion** ** (*n* = 828)**	***P*-value**
SR index on admission, mean (SD)	0.542 (0.104)	0.536 (0.106)	0.544 (0.104)	
SR index on follow-up, mean (SD)	0.515 (0.101)	0.487 (0.098)	0.524 (0.101)	
SR index change, mean (SD)	−0.027 (0.073)	−0.049 (0.090)	−0.020 (0.065)	^*^
**Longitudinal axis type of hematoma on admission**, ***n*** **(%)**				
AP	708 (64.7)	175 (65.8)	533 (64.4)	
LR	60 (5.5)	9 (3.4)	51 (6.2)	
SI	213 (19.5)	55 (20.7)	158 (19.1)	
NL	113 (10.3)	27 (10.2)	86 (10.4)	
**Diameters of hematoma on admission, mean (SD), mm**				
Length (AP)	60.54 (23.3)	64.1 (25.0)	59.4 (22.6)	^*^
Width (LR)	43.9 (15.0)	46.9 (17.2)	43.0 (14.1)	^*^
Height (SI)	55.3 (15.8)	58.8 (16.6)	54.2 (15.4)	^*^
**Length change of hematoma diameters, mean (SD), mm**				
AP	2.8 (9.8)	4.8 (13.0)	2.1 (8.5)	^*^
LR	1.9 (9.0)	2.8 (11.3)	1.6 (8.1)	^*^
SI	−0.5 (9.2)	−1.2 (16.8)	−0.4 (4.7)	^*^
Distance of center movement, mean (SD), mm	3.5 (5.4)	6.1 (8.2)	2.7 (3.7)	^*^

* *P < 0.05. AP, anterior-posterior; LR, left-right; SI, superior-inferior; NL, no longitudinal axis; SD, standard deviation; SR, surface regularity*.

**Figure 2:**
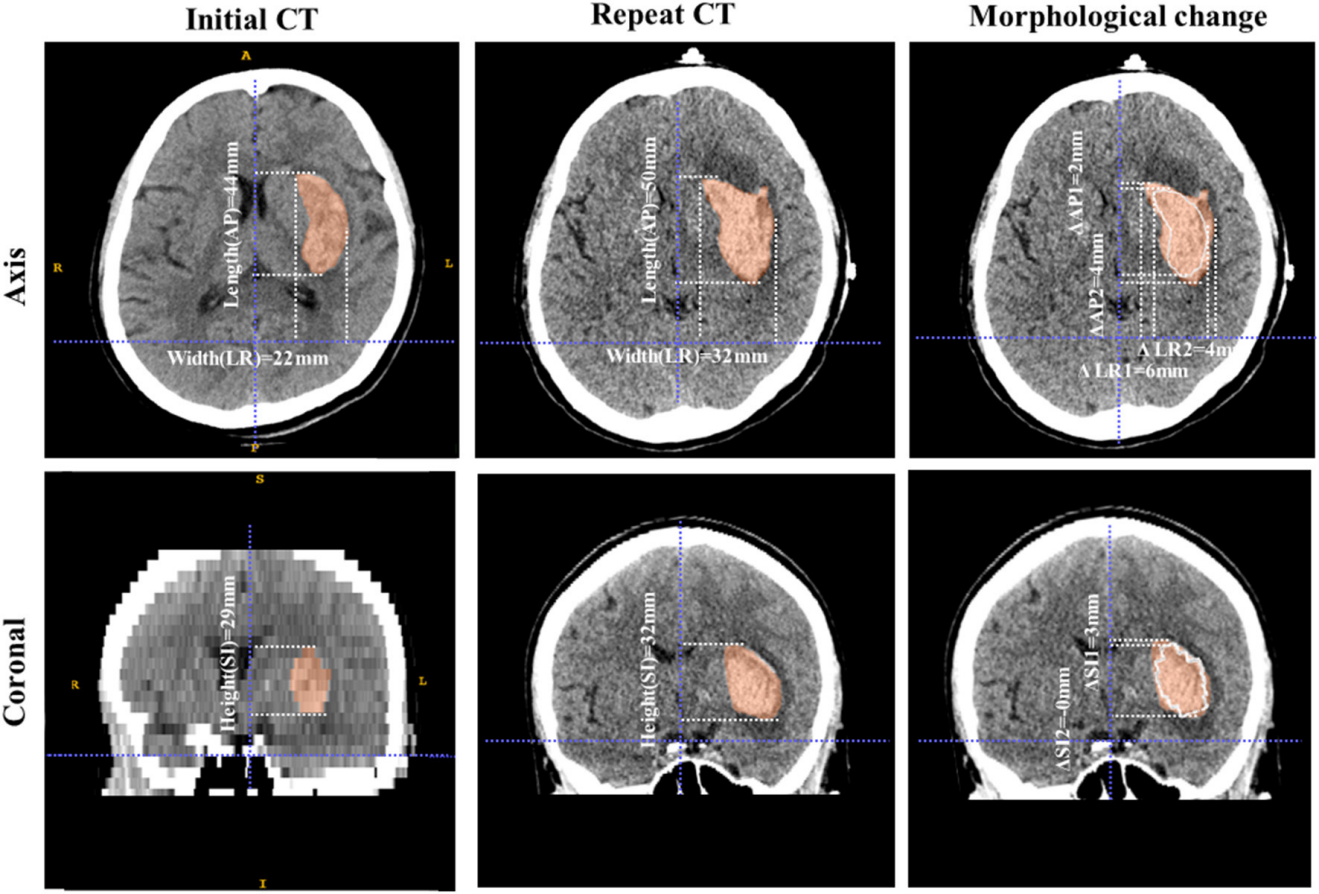
Example of morphological change in a hematoma. These images are from a 73-year-old male patient with ICH. The volume of the hematoma was 15.6 mL on initial CT and 23.5 mL on repeat CT. The first column shows the shape characteristics of the initial hematoma, including its three diameters (length 44 mm, width 22 mm, and height 29 mm). The longitudinal axis is of the AP type and the SR index is 0.628. The second column shows the shape characteristics of the hematoma on the repeat scan, including its three diameters (length 50 mm, width 32 mm, and height 32 mm). The longitudinal axis is of the AP type and the SR index is 0.552. The third column shows the morphological change. The white line is the contour of the initial hematoma and the red area is the hematoma as of the repeat scan. The length changes of the hematoma diameters are 6 mm, 10 mm, and 3 mm in the AP, LR, and SI directions, respectively. The direction change of the hematoma diameters is LR and the longitudinal axis type does not change (AP). The SR index decreases, which means that the hematoma becomes more irregular from initial CT to repeat CT.

The most common type of longitudinal axis was the AP direction (64.7%), followed by the SI direction (19.5%). A total of 10.3% of hematomas had an approximately spherical shape and were categorized as the NL type in this study. There was no significant difference in the longitudinal axis types between the expansion and non-expansion groups (Table 2). The length change of the diameters was small, and the largest change was in the AP direction, with an increase of 4.8 mm in the expansion group (Figure 2). The distance of center movement was small, with a median of 3.5 mm for all patients and 6.1 mm in the expansion group.

We investigated the patterns of change in the diameters and geometric center for different longitudinal axis types (Table 3). Regardless of the longitudinal axis type, the diameter direction change was mostly AP [43.1% (471/1094) in total, ranging from 38.3 to 45.2%], followed by LR (ranging from 23.9 to 31.0%). The length change of the diameters was <3.3 mm in most cases.

**Table 3:** Morphological changes by longitudinal axis type.

	**Hematoma longitudinal axis type**			
	**AP (*n* = 708)**	**LR (*n* = 60)**	**SI (*n* = 213)**	**NL (*n* = 113)**
**Direction change of hematoma diameters**, ***n*** **(%)**				
AP	320 (45.2)	23 (38.3)	83 (39.0)	45 (39.8)
LR	197 (27.8)	16 (26.7)	66 (31.0)	27 (23.9)
SI	88 (12.4)	12 (20.0)	21 (9.9)	21 (18.6)
No direction change	103 (14.5)	9 (15.0)	43 (20.2)	20 (17.7)
**Length change of hematoma diameters, mean (SD), mm**				
AP	3.0 (10.5)	1.8 (6.7)	2.2 (7.4)	3.3 (10.9)
LR	1.8 (8.2)	0.4 (4.0)	2.9 (13.2)	0.9 (5.1)
SI	−0.3 (6.9)	−0.8 (4.4)	−0.6 (6.4)	−1.7 (21.0)
Distance of center movement, mean (SD), mm	3.6 (3.5)	2.3 (2.0)	3.4 (7.0)	4.1 (10.4)

*AP, anterior-posterior; LR, left-right; SI, superior-inferior; NL, no longitudinal axis; SD, standard deviation*.

**Figure 3:**
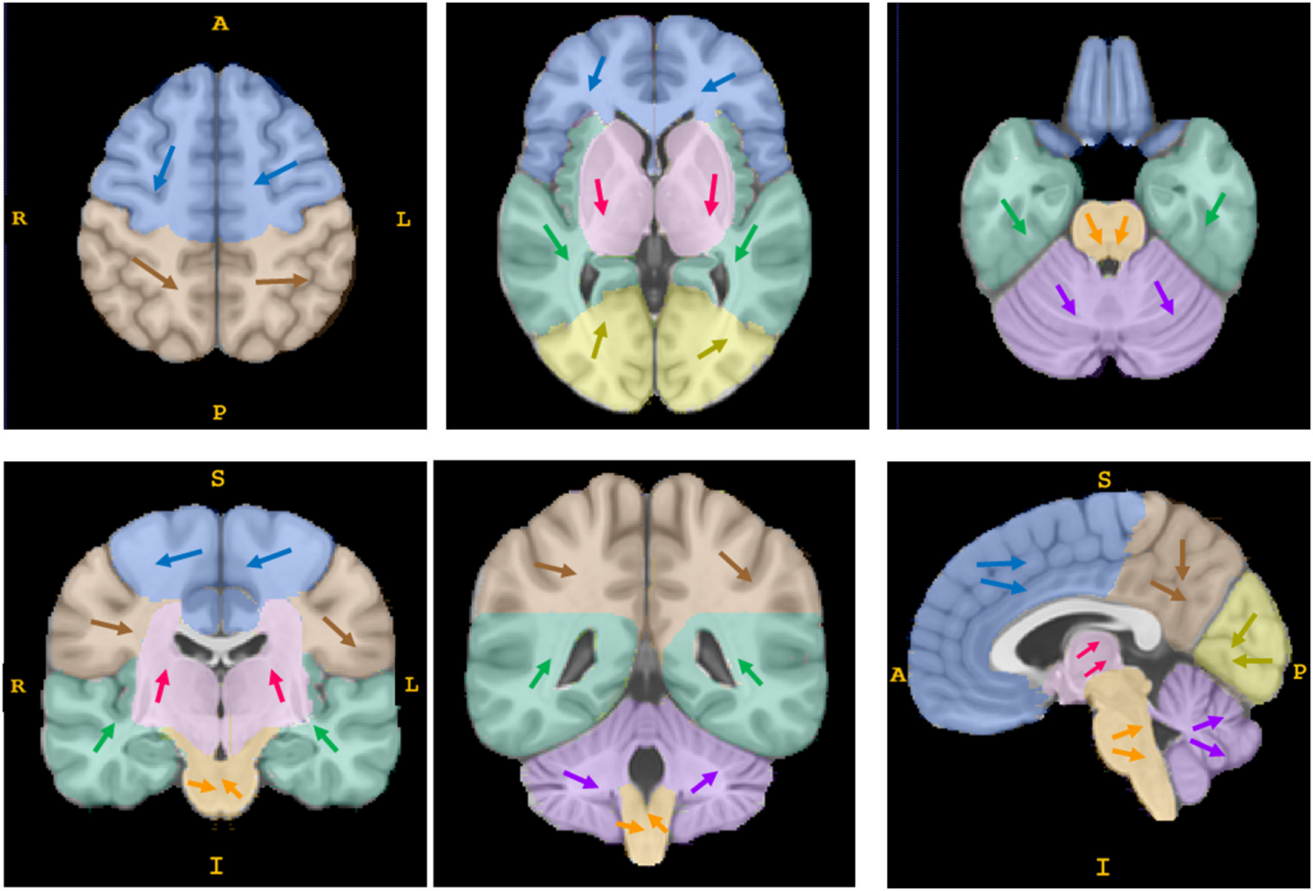
The direction of movement of the geometric center of hematomas. Each arrow of a different color represents the synthesized direction of center movement in a different anatomical region (the frontal lobe is shown in blue, the parietal lobe in brown, the insula and temporal lobe in green, the occipital lobe in yellow, the basal ganglia/thalamus area in pink, the brain stem in orange, and the cerebellum in purple). The direction of center movement in deep supratentorial regions (basal ganglia/thalamus area) and subtentorial regions (brain stem and cerebellum) was in the direction of gravity as patients lay in a supine position; some supratentorial lobar hematomas showed no such pattern. This is a schematic diagram; the details are shown in attachment 1.

The distance of center movement was small, ranging from 2.3 to 4.1 mm. As Figure 3 shows, the direction of center movement in deep supratentorial hematomas was in the direction of gravity as patients lay in a supine position. A similar pattern was also observed in subtentorial hematoma; however, it did not exist in some supratentorial lobar hematomas, such as those in the frontal lobe, parietal lobe and occipital lobe (Figure 3, Attachment 1).

Although most changes in diameter length were small, there were 139 cases with obvious changes, where the changes in diameter length and the distance of center movement were both greater than 10 mm, or the direction change was inconsistent with the longitudinal type. However, among these cases with obvious changes, only 53 (38.1%) patients' longitudinal axis types were changed, accounting for 4.84% of all patients (53/1,094).

**Figure 4:**
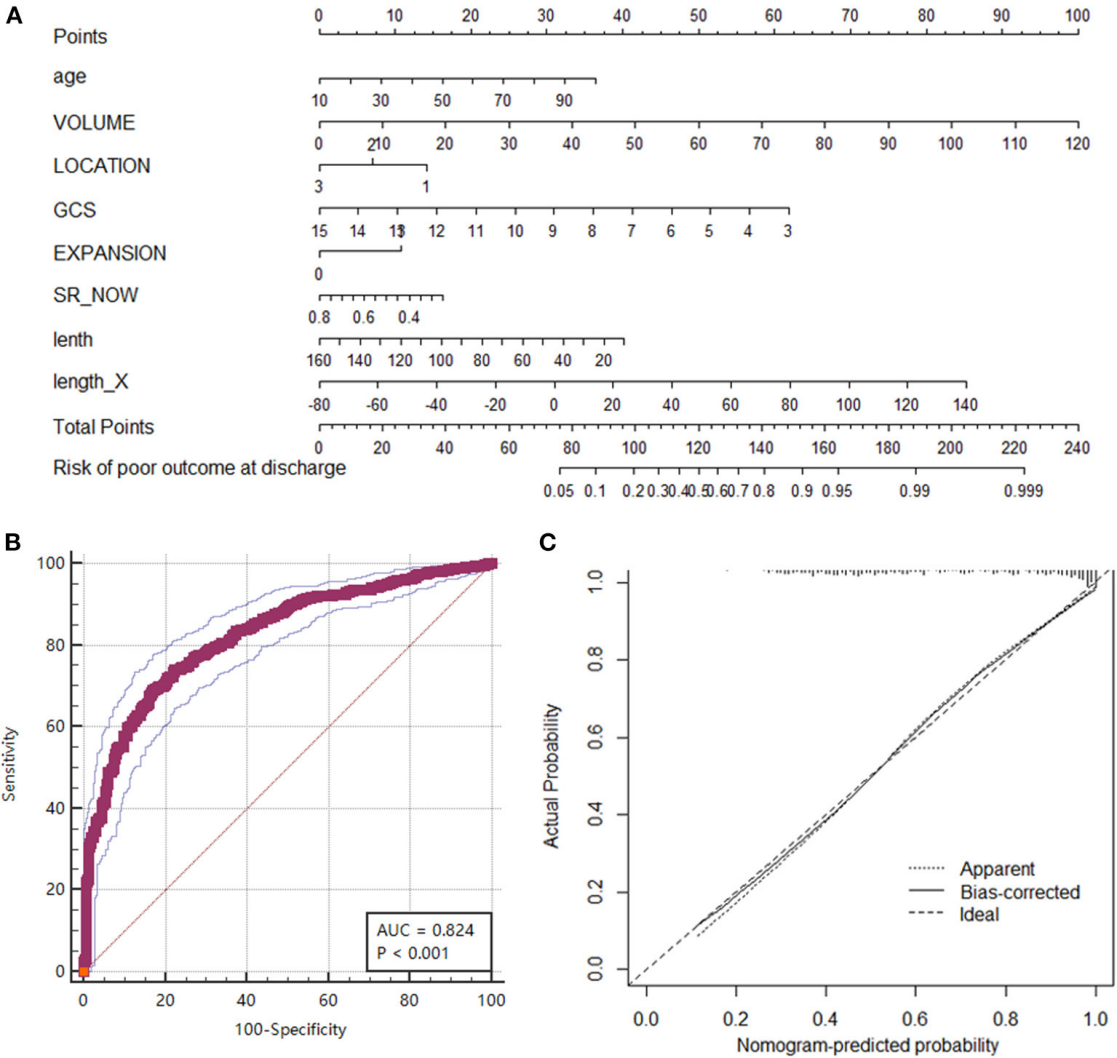
Nomogram of the prognostic model for predicting poor outcomes (GOS ≤ 3) at discharge. **(A)** The nomogram was developed from a multivariable logistic regression model based on age, volume, location, GCS, hematoma expansion, initial SR index, diameter lengths, and length change in the LR direction. **(B)** ROC curve of the nomogram representing the discrimination performance of the model. **(C)** Calibration curve of nomogram. A calibration curve depicts the calibration of a model in terms of the agreement between the predicted risk of a poor outcome and the outcome actually observed. The Y-axis represents the actual poor-outcome rate. The X-axis represents the predicted poor-outcome risk. The diagonal dotted line represents a perfect prediction by an ideal model. The solid black line represents the performance of the nomogram, where a closer fit to the diagonal dotted line represents a better prediction.

To explore the clinical significance of the morphological change in HE, we established a prognostic nomogram to predict poor outcomes (GOS ≤ 3). Eight potential predictors (age, volume, location, GCS, hematoma expansion, initial SR index, hematoma diameter length, and length change of the LR diameter) were selected from 19 collected variables by using multivariate logistic regression (Supplementary Table 2). Then, the logistic regression analysis was visualized as a nomogram (Figure 4A), which was preliminarily built to predict the probability of poor outcome in ICH patients. ROC curve analysis indicated that the nomogram performed well in prognostic prediction, with an AUC of 0.824 (95% CI 0.800, 0.846). The calibration plot also showed excellent agreement between the nomogram predictions and actual observations in ICH patients with GOS ≤ 3 (Figure 4). In particular, the length change of the LR diameter (the lateral expansion) was a morphological change factor that contributed strongly to the model, with an odds ratio of 1.1386 (95% CI: 1.0216, 1.2691). All these findings suggested that our prediction model including morphological change parameters had good performance in predicting poor clinical outcomes in ICH patients. This scale should remind physicians to pay attention to lateral expansion, especially in ICH patients who are predicted to have GOS ≤ 3.

## Discussion

In this study, we investigated the patterns of morphological change in sICH based on a large cohort. We found that the initial hematoma tended to be more irregularly shaped, with a larger SR index, than the developed hematoma. In deep supratentorial hematomas and subtentorial hematomas, the direction of center movement was toward the pull of gravity. Most hematomas had their longitudinal axis in the AP direction (64.7%), and the direction of the diameter change was AP in approximately 40% of patients. The length change of the diameters and the distance of center movement were <4 mm. The longitudinal axis type did not change between the initial and repeat CT scans in most patients. In addition, one morphological change parameter, the length change of the diameter in the LR direction (lateral expansion), was found to be associated with poor prognosis in ICH patients. The prediction model including lateral expansion for ICH patients with poor prognosis showed good performance. The results of our analysis provide a new perspective on hematoma expansion in terms of morphological changes. Physicians should take these results as a reminder not to ignore ICH with lateral expansion.

The SR index quantifies irregularity by the ratio of surface area to volume (20, 21). As in previous studies (21), the SR index decreased in the repeat hematoma group, especially in the expansion group, which indicated that the hematomas tended to become more irregular as they developed. Previous studies showed that the efficacy of hematoma evacuation surgery decreased in irregular hematoma (31), and our findings reminded the surgeon to consider the tendency of hematoma to be more irregular before making surgical decisions.

Similar to previous studies (11–13, 22), our study showed that hematoma growth was asymmetric and that the geometric center moved in HE. Furthermore, we found a pattern regarding center movement. In subtentorial and deep supratentorial hematomas, the center tended to move in the direction of gravity. Although the center moved only a short distance, researchers should take this movement as a reminder to consider the effect of gravity when studying the pathophysiological mechanism of acute-phase hematoma formation and expansion.

In this study, we found that the longitudinal axis type did not change in 90% of HE cases. Although the direction change of the diameters did not always align with the longitudinal axis, the length changes of the diameters were ordinarily <4 mm, which is insufficient to change the longitudinal axis type. The median distance of center movement was only 3.5 mm, such that the change would not influence the drainage trajectory traversing the epicenter of the hematoma (31, 32). These findings alleviate the concern as to whether the longitudinal direction or geometric center would change after drainage surgery for HE (33, 34).

Another interesting finding in our study was that lateral expansion (the length change of the diameter in the LR direction) was associated with poor outcomes. As important factors in HE and prognosis, the shape features of the initial hematoma have been described by various methods and demonstrated to be associated with outcomes. These studies have included qualitative analytical variables, such as Barras grade, island sign and satellite sign (8, 14, 20), and quantitative analytical variables, such as the SR index, compactness, Fourier factor and fractal dimension (15, 21, 35). The present study is the first to relate changes in hematoma morphology to the patient's prognosis. The prediction model showed that the risk of a poor outcome increased by a factor of 1.139 for every 1 mm of lateral expansion. This finding should remind physicians not to ignore lateral expansion, especially in ICH patients who are predicted to have poor outcomes.

This study has several limitations. For the purposes of maintaining analytic rigor, we excluded patients who had undergone any invasive operation before repeat CT, as these interventions directly affect hematoma shape. We also excluded patients with time intervals of <8 h or more than 72 h between scans, as the shape of a hematoma tends to be most stable from 8 to 72 h. The volume of the lesion may significantly increase in the first 8 h, and absorption starts after 72 h (34). These criteria may bias our sample toward populations less severely affected by their hemorrhage. In addition, the absolute value of the length change was affected by the accuracy of the registration process. Although we strictly excluded failed registration (36) and manually checked each case, there might still have been some registration error. Considering that the actual length change was small, this registration error may have an adverse effect on the accuracy of the calculated absolute value of the length change. Moreover, the longitudinal axis in our study was defined as the longest of the three diameters. These diameters were measured parallel to the coordinate system to compare the direction changes across different hematomas (Supplementary Figure 1). Thus, the longitudinal axis as defined here is merely the projection of the actual longitudinal axis onto the coordinate axis that best approximates its direction. This imperfect definition might limit the interpretability of the findings. Finally, while our study included more than one thousand patients who underwent repeat imaging, all the patients were from a single country, China. The retrospective nature of this multicenter analysis is another limitation. Further validation must be carried out with independent data sets to ensure generalizability.

In conclusion, the present study provides a morphological perspective on hematoma expansion by a novel approach. We identified certain patterns of morphological change in HE. As hematomas enlarged, they shifted in the direction of gravity and tended to be more irregular. The most common longitudinal axis type of hematoma was AP, which did not change during HE. Based on our findings, we used morphological change parameters to establish a novel, promising prognostic nomogram model for the individualized prediction of poor outcomes in ICH patients. This nomogram requires further validation in other centers.

## Data Availability Statement

The raw data supporting the conclusions of this article will be made available by the authors, without undue reservation.

## Ethics Statement

The studies involving human participants were reviewed and approved by Institutional Review Board of Peking Union Medical College Hospital. Written informed consent for participation was not required for this study in accordance with the national legislation and the institutional requirements.

## Author Contributions

CJB and XT were the project lead for the current study and contributed equally to the study. CY, WXia, ZQ, YZ, WXin, TF, and CJB collected and extracted data. CJB, XT, and SH conducted data and statistical analysis. CJB wrote the manuscript. FM, MW, and WJ provided significant advice about the manuscript. WR and JY reviewed and revised the manuscript. WR, FM, and JY are co-corresponding authors. All authors read and approved the final manuscript.

## Funding

This study has received funding by National Key R&D Program of China (2018YFA0108603), Chinese Academy of Medical Sciences (CAMS) Innovation Fund for Medical Science (2020-I2M-C&T-B-031 and 2020-I2M-C&T-B-028), Beijing Tianjin Hebei basic research cooperation project [19JCZDJC64600 (Z)], and Huazhong University of Science and Technology Union Shenzhen Hospital Fund (NS202001).

## Conflict of Interest

JY, XT, SH, and WXia are employee of Tencent AI Lab. The remaining authors declare that the research was conducted in the absence of any commercial or financial relationships that could be construed as a potential conflict of interest.

## Publisher's Note

All claims expressed in this article are solely those of the authors and do not necessarily represent those of their affiliated organizations, or those of the publisher, the editors and the reviewers. Any product that may be evaluated in this article, or claim that may be made by its manufacturer, is not guaranteed or endorsed by the publisher.
