# Supplementary material for: The Patterns of Morphological Change During Intracerebral Hemorrhage Expansion: A Multicenter Retrospective Cohort Study
Source: Front Med (Lausanne). 2022 Jan 13;8:774632. doi: 10.3389/fmed.2021.774632 (PMC8792842; doi:10.3389/fmed.2021.774632)
Supplement: Supplementary file 1 [file Data_Sheet_1.docx]

Supplementary Material

# Supplementary Figures and Tables

**Supplementary Table 1** Overview of datasets

| Number | Cohort 1 | Cohort 2 | Cohort 3 | Cohort 4 |
| --- | --- | --- | --- | --- |
| Hospital code | LYRM | DGRM | ZQRM | QHRM |
| Scanner | SIEMENS, GE | SIEMENS, GE, PHILIPS, TOSHIBA | SIEMENS | GE |
| Number of patients included | 367 | 128 | 426 | 173 |
| Summary | Eligible NCCT scans for training and internal tests from Jan 1, 2016, to Aug 30, 2020, at Linyi People’s Hospital. | Eligible NCCT scans for training and internal tests from Dec 1, 2017, to Jun 30, 2020, at Dongguan People’s Hospital. | Eligible NCCT scans for external tests from Jan 1, 2017, to May 31, 2020, at Zhangqiu People’s Hospital. | Eligible NCCT scans for external tests from Jan 1, 2019, to Jun 30, 2020, at Qinghai Provincial People's Hospital. |
| Hospital level | Secondary care center | Secondary care center | Secondary care center | Tertiary referral center |
| Time interval between CT scans, median (IQR), hr | 23.37 (16.12 - 37.64) | 23.28 (14.86 - 41.28) | 21.95 (15.17 - 27.33) | 24.05 (18.41 - 42.31) |
| Time interval between CT scans, n (%) |  |  |  |  |
| 8h – 24h | 196 (53.4) | 68 (53.1) | 257 (60.3) | 86 (49.7) |
| 24h – 48h | 125 (34.1) | 36 (28.1) | 137 (32.2) | 63 (36.4) |
| 48h – 72h | 46 (12.5) | 24 (18.8) | 32 (7.5) | 24 (13.9) |

**Supplementary Table 2** Multivariable logistic regression model for predicting poor outcomes (GOS≤3) at discharge in intracerebral hemorrhage patients based on clinical characteristics and hematoma morphology.

| Variable and intercept | β* | Odds ratio (95% CI) | *P* value |
| --- | --- | --- | --- |
| Age | 0.0272 | 1.6303 (1.3203, 2.013) | <0.0001 |
| Volume | 0.0558 | 4.1273 (2.6792, 6.358) | <0.0001 |
| Location | -0.4726 | 0.38857 (0.24102, 0.62645) | 0.0001 |
| GCS | -0.3456 | 0.25101 (0.18612, 0.33852) | <0.0001 |
| Hematoma expansion | 0.7207 | 2.0558 (1.3994, 3.02) | 0.0002 |
| Initial SR index | -1.9835 | 0.72223 (0.5339, 0.97701) | 0.0348 |
| Hematoma diameter length | -0.0180 | 0.57315 (0.37838, 0.86819) | 0.0086 |
| Length change of LR diameter | 0.0260 | 1.1386 (1.0216, 1.2691) | 0.0190 |
| Intercept | 4.8261 |  |  |

*β: the regression coefficient; CI confidence interval; GCS Glasgow Coma Score; SR surface regularity; LR, left-right.

**Supplementary Figure 1** Method for determining diameters. The case was a 42-year-old man in Cohort 1 who had a 27.5 mL hematoma. The length (51 mm) and width (31 mm) were determined on the axial hematoma slice with the maximum area (Fig. C). The height (40 mm) was determined in the coronal plane (Fig. D). The measurement process was automatically performed in 3D space by an algorithm (Fig. A, Fig. B) after the data were registered to an atlas.


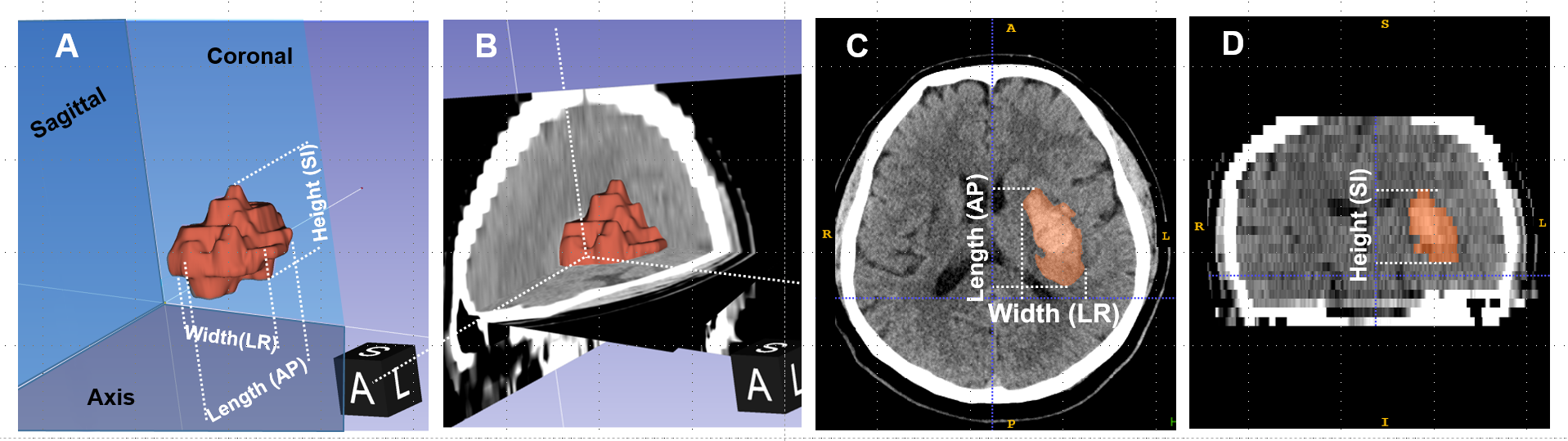


**Supplementary Figure 2** Four longitudinal axis types. Based on which axis had the longest diameter, the longitudinal axis of each initial hematoma was categorized as one of four types: AP (Fig. A), LR (Fig. B), SI (Fig. C) and no longitudinal axis (NL) (Fig. D).


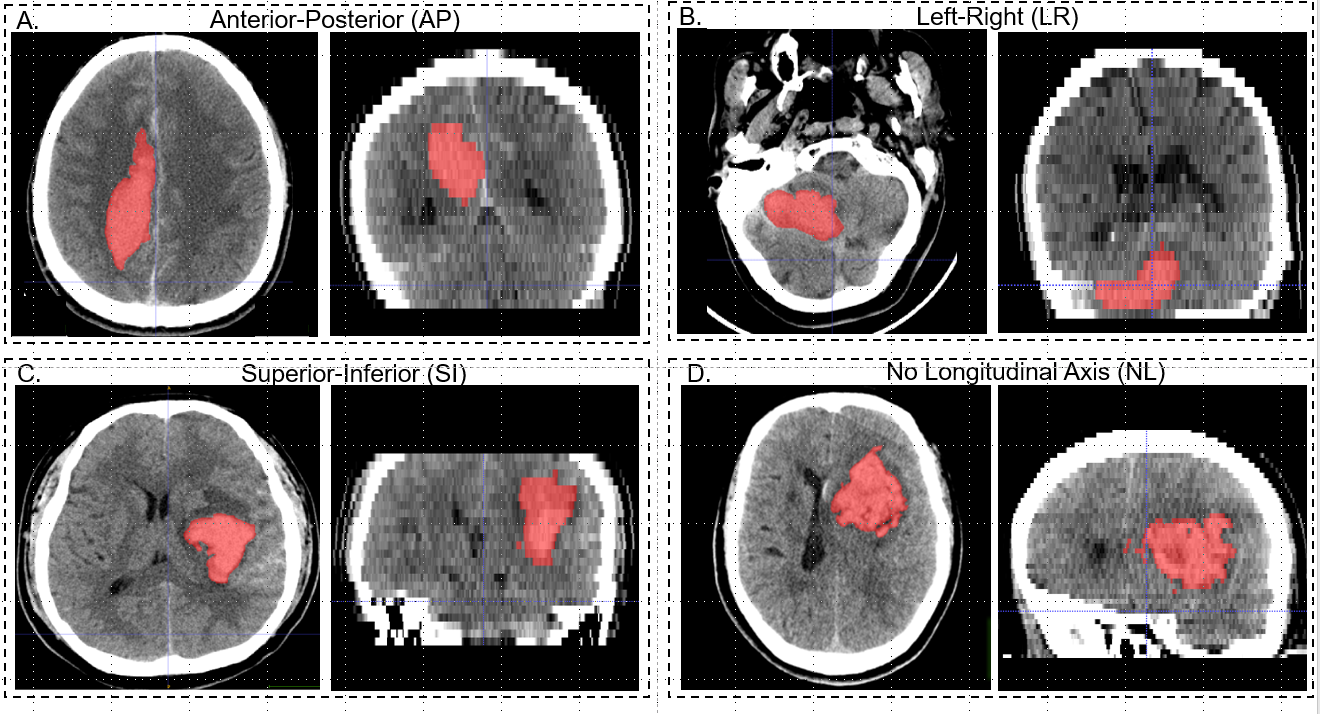


**Supplementary Figure 3** Example of the distance and direction of center movement. The first row represents an example case. The red arrow in the 3D column shows the distance and direction of center movement between center 1 (initial CT) and center 2 (repeat CT). The last three figures in the first row show the projection of the center movement direction in the standard planes (axial, coronal and sagittal). The second row represents an example of synthesis. The dark blue arrow shows the direction of synthesized center movement based on the center movement of all hematomas located in the left basal ganglia/thalamus area. The sky blue arrow shows the direction of synthesized center movement for hematomas located on the right side. In the last three images in the second row, the red arrows represent the direction of center movement in each case, and the blue arrows represent the projection of the synthesized direction in the three standard planes, visualized according to one atlas. Details of the other synthetic arrows are shown in attachment 1.


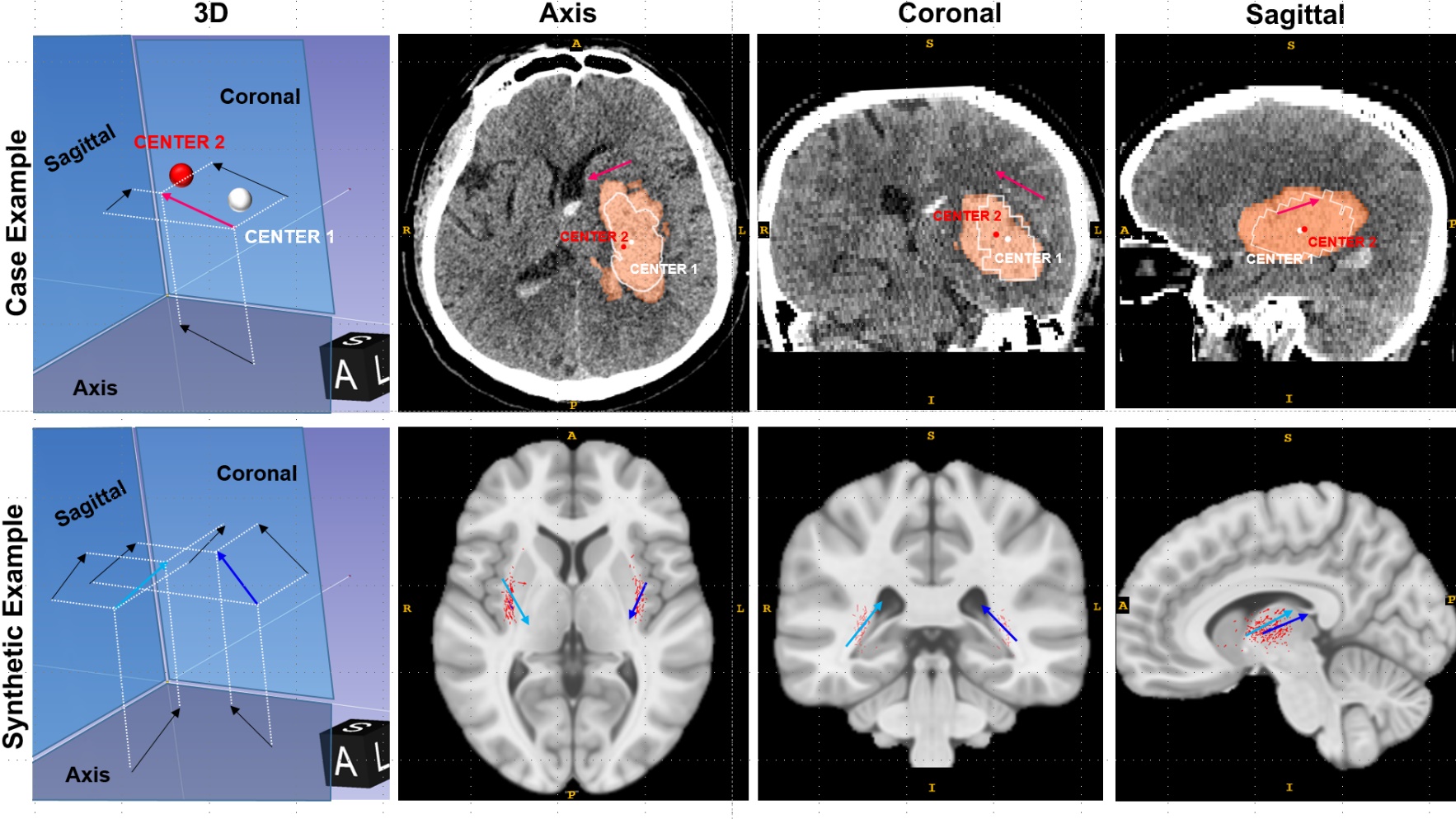


**Supplementary Figure 4** ICH location prevalence as shown after registration to an atlas. The proportion of patients with ICH involving each voxel is represented in a 3D histogram (the right side of image represents the left side of the brain) overlaid on an MRI T1 atlas. The hematomas were mainly distributed in the basal ganglia/thalamus area, and there were more hematomas on the left side than on the right side.

**
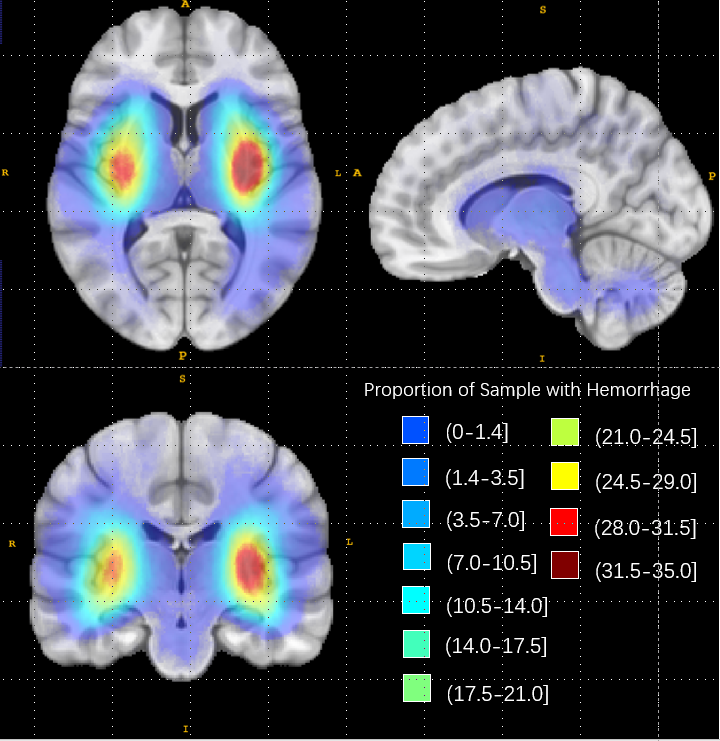
**
